# Research Progress and Prospects of Intelligent Measurement and Control Technology for Tillage Depth in Subsoiling Operations

**DOI:** 10.3390/s25123821

**Published:** 2025-06-19

**Authors:** Yue Deng, Wenyi Zhang, Bing Qi, Yunxia Wang, Youqiang Ding, Haojie Zhang

**Affiliations:** Nanjing Institute of Agricultural Mechanization, Ministry of Agriculture and Rural Affairs, Nanjing 210014, China; 82101235635@caas.cn (Y.D.); zhangwenyi@caas.cn (W.Z.); qibing@caas.cn (B.Q.); dingyouqiang@caas.cn (Y.D.); 821012430114@caas.cn (H.Z.)

**Keywords:** soil mechanical resistance detection, tillage depth measurement, tillage depth regulation

## Abstract

Deep tillage is a conservation tillage method that aims to break the plow pan layer. It provides significant benefits, including enhanced root development, improved soil quality, and substantial increases in crop yields. The depth of tillage is a crucial factor in assessing the effectiveness of deep tillage operations. Accurate regulation of tillage depth in deep tillage equipment is vital for ensuring the high-quality and efficient execution of these practices. The distribution of mechanical resistance within the soil can effectively indicate the location of the plow pan layer and serves as the main reference for setting the tillage depth for machinery. This paper examined the current state of research on tillage depth control technology for deep tillage operations. It focused on three main technical areas: soil mechanical resistance detection, tillage depth measurement, and tillage depth regulation. The report discussed the working principles of various technologies and compared the existing methods. Additionally, the paper analyzed the challenges faced in the development of tillage depth control technology in China and offers recommendations for future advancements. It highlighted that leveraging information and digital technologies to determine the distribution of the soil plow pan layer, along with the integration of efficient and intelligent control technologies for precise tillage depth regulation, represented a key direction for the future development of deep tillage operations.

## 1. Introduction

The plow pan is a hard soil layer formed in the soil due to the long-term use of single tillage methods such as rotary tillage and tillage, coupled with the rolling of production machines and tools during field operations [1]. The research showed that the plow pan is widespread in dry farming areas in China. Above the plow pan is the topsoil layer that is cultivated all the year round, and below the plow pan is the core soil layer that is not cultivated, but the soil is relatively soft, forming an unreasonable plough layer structure of “shallow effective plough layer and compacted plow pan”. Because the bulk density of the plow pan is far greater than the reasonable index suitable for crop growth, it will seriously hinder the growth of crop roots and cause yield reduction [2]. Subsoiling is a conservation tillage method with the purpose of breaking the bottom layer of plow. After subsoiling, crop roots can easily grow downward through the original bottom layer of plow. Compared with rotary tillage and tillage, subsoiling has obvious advantages in improving soil and increasing crop yield [3,4]. In order to improve the quality of cultivated land and promote the sustainable development of agriculture, subsoiling technology has been highly valued in China.

Subsoiling depth is an important index to evaluate the quality of subsoiling operations [5], and it is also the main basis for the government to subsidize. The implementation of subsoiling operations is mostly based on agricultural machinery cooperatives and large agricultural machinery households, and subsidies are provided according to whether the subsoiling depth reaches the set unified ploughing depth.

However, most of China’s production areas have not achieved large-scale operation, and the land area is small, and the distribution is not concentrated. The frequency of tillage machinery and the rolling position used in different plots are different, which leads to great differences in the distribution position and thickness of the bottom layer of plough in different production fields [6]. Therefore, the subsoiling operation with a unified ploughing depth has the following problems: Compared with the position of the plow pan, if the ploughing depth is too small, the plow pan cannot be broken, and the effect of improving the soil cannot be achieved. If the ploughing depth is too large, the increase in subsoiling resistance will increase the power consumption of the tractor, increase the operating cost, and reduce the energy consumption efficiency.

In view of the complexity and spatiotemporal variability of field environments, the regulation of tillage depth often requires multiple rounds of parameter iteration to achieve stable and consistent operational effects. Topographic undulations, spatial heterogeneity of soil texture, and differentiated crop growth requirements collectively impose diversified demands on tillage depth control strategies. To address these challenges, researchers have shifted their focus to real-time online regulation technologies for tillage depth, with current studies primarily encompassing two technical approaches: whole-machine tillage depth control and row-independent depth regulation.

Tillage machinery can be categorized into suspended and traction operations based on the attachment mode of implements during tractor operation, with significantly different tillage depth control strategies corresponding to each category.

In recent years, countries with extensive hilly and mountainous terrains such as China, Japan, and South Korea have predominantly focused tillage depth regulation research on suspended implements. By contrast, studies on tillage depth control for suspended tools in Europe and America are relatively limited, while China has established a more systematic research framework in this field. Depth control of domestic suspended implements is primarily realized through the hydraulic suspension system of tractors, which can be subdivided into height control, position control, force control, slip rate control, torque control, and multi-parameter integrated control modes according to control variables.

In plain areas suitable for large-scale mechanized operations, developed agricultural countries in Europe and America commonly adopt combined soil preparation machines for high-power traction operations, whose tillage depth regulation typically relies on variable rate control technology based on soil compaction prescription maps to achieve precise whole-machine depth control.

Compared with traditional regulation modes based on unified tillage depth thresholds, the dynamic tillage depth regulation technology based on prescription maps represents a more efficient intelligent operation approach by achieving adaptive adjustment of subsoiling depth through precise identification of plow pan spatial distribution characteristics. The spatial localization of plow pans in the tillage layer is primarily based on the gradient variation of soil mechanical resistance, where regions with abrupt resistance jumps are identified as plow pan interfaces. Therefore, intelligent tillage depth control necessitates real-time sensing of key parameters such as soil mechanical resistance, with soil mechanical force acquisition methods classified into two technical systems: static penetration testing and dynamic response measurement.

This paper provided an overview of the research advancements in tillage depth measurement and control technologies, both globally and within China. It outlined the current status of three major areas: soil mechanical resistance detection, tillage depth measurement, and tillage depth control.

This paper categorized and evaluated the different methods used in these areas according to their operational principles, offering a comparative analysis. It also examined the current challenges faced in the field of tillage depth measurement and control. Based on this assessment, we discussed potential future trends for tillage depth control that were suitable for deep tillage practices in China, aiming to offer insights and guidance for the accurate application of deep tillage technology.

## 2. Status of Soil Mechanical Resistance Measurement

Soil mechanical resistance refers to the force that is generated when various agricultural machinery, such as subsoilers, engage with the soil. During the cultivation process, it is the soil that undergoes multiple types of deformation, including compression, torsion, and shearing, due to the action of the subsoiler’s blade. The reaction of the soil against the farming machinery is known as soil deformation resistance. When the soil is worked by the subsoiler blade, the structure of the cohesive soil is caused to separate and break apart, resulting in cutting resistance. As the subsoiler moves through the soil, internal friction is generated between the soil and the subsoiler. Additionally, during cultivation, it is the soil in the top layer that undergoes displacement relative to the soil in the lower layers, which also generates resistance [7]. All these factors collectively constitute soil mechanical resistance. Soil mechanical resistance is one of the physical and mechanical properties of soil and is strongly correlated with soil strength [8].

The two key soil mechanical parameters essential for assessing soil mechanical resistance are cohesion and the internal friction angle. One method that can be employed for this assessment is the triaxial shear test. However, obtaining hollow cylinders that are twice as long as the cultivation depth is challenging, and the spatial variability in the distribution of the plow layer adds another layer of difficulty. This approach is not only time-intensive and expensive but also has inherent limitations, as it does not adequately account for the spatial variability of the plow layer. Furthermore, the necessity for sampling means it cannot fulfill the requirements for the real-time, online measurement of soil mechanical resistance.

There are several methods available for measuring soil mechanical resistance, encompassing the non-continuous measurement approach, telemetry, indirect testing, and the continuous measurement method in Table 1.

### 2.1. Discontinuous Measurement Method

When measuring soil mechanical resistance, the two key parameters are cohesion and the angle of internal friction. Tollner et al. [9] had created a computer-controlled cone penetrometer capable of measuring the cone index and stress relaxation. While the triaxial shear test was another option, it presented challenges such as the difficulty in obtaining hollow cylinders that were twice the length of the tillage depth and the spatial variability of the plow layer.

Discontinuous measurement methods exhibit remarkable flexibility and adaptability, allowing for the flexible selection of measurement points in complex terrains such as hilly areas and terraced fields. This approach remains unaffected by mechanical traversal routes, landform undulations, or crop obstruction. It enables targeted static measurements of localized soil zones, such as crop root zones and plow layers, where spatial heterogeneity necessitates focused analysis. By conducting repeated measurements at discrete points, this method enhances the reliability and accuracy of localized data, avoiding external interferences that cause data fluctuations. Therefore, discontinuous measurement data can serve as a benchmark for calibrating continuous monitoring systems.

This method was not only time-intensive and expensive but also had inherent limitations. It struggled to accommodate the spatial heterogeneity of the plow layer and cannot deliver real-time data. The primary disadvantage of using a discontinuous measurement for mechanical resistance was that it was static and cannot be applied to continuous or ongoing measurements, thus failing to meet the requirements for online monitoring.

### 2.2. Telemetry Method

Neher-Neumann et al. [10] had explored the use of soil dielectric loss to measure soil mechanical resistance. However, the results were highly variable, allowing only for the determination of general trends rather than precise values.

Luo et al. [11] had employed the microwave reflection effect. By transmitting microwaves and capturing the reflected waves, they measured the mechanical resistance of the soil. Their experiments revealed that in a semi-logarithmic coordinate system, the microwave reflection loss of the soil decreases linearly as the cone index increases.

Telemetry calculates soil mechanical resistance through microwave or spectral methods, featuring fast measurement speed and reducing the damage to field soil caused by agricultural machinery in discontinuous and continuous measurement methods.

However, due to soil complexity, this method is affected by numerous uncertain factors, and the accuracy of results is constrained by variables such as vegetation, weather, and temperature. Currently, it remains at the theoretical research stage, only conducted on soil samples in laboratories, and is not yet ready for field production applications.

### 2.3. Indirect Measurement Method

Koostra et al. [12] had created an air permeability sensor to indirectly assess soil mechanical resistance. This technique involves “introducing” air into the soil and measuring the backpressure while ensuring a consistent flow rate. A probe with air outlets was attached to the soil, forming a contact surface. A mass flow controller managed the airflow, which was then directed to the probe through an adjustable handle. The probe itself was a thin vertical plate with air outlets on both sides. As the plate was drawn through the soil, the airflow is directed vertically into the soil at the point where the plate meets the soil. The air pressure downstream of the mass flow controller was measured using a pressure sensor, allowing for the indirect calculation of soil mechanical resistance.

The indirect measurement method predicts the cone index and quantifies soil mechanical resistance by measuring various parameters that do not disrupt soil structure. Its core lies in excavating the intrinsic correlations between multiple parameters and soil mechanical resistance, so as to achieve more accurate determination of soil mechanical resistance.

### 2.4. Continuous Measurement Method

The continuous measurement method typically refers to a technique that acquires soil mechanical resistance by continuously collecting the force applied to soil-cutting devices or blades during the soil cutting process. This approach enables the continuous measurement of soil mechanical resistance with high sampling efficiency.

Adamchuk et al. [13] developed a vertical blade fitted with an array of strain pressure sensors, which was attached to a tractor. This system enabled the continuous measurement of soil mechanical resistance at various depths. The correlation coefficient between the soil mechanical resistance values at the same depth, as measured by this method, and those obtained using the static discontinuous method with a cone penetrometer, was 0.95.

Sirjacobs et al. [14] positioned strain gauge pressure sensors between the tractor and the subsoiler support base. This setup allowed for the measurement of the horizontal resistance, vertical resistance, and torque exerted on the subsoiler support base, effectively recording the soil’s mechanical resistance. Additionally, this method marked the beginning of utilizing an analog-to-digital converter and a microcomputer system to gather soil mechanical resistance data continuously and in real time.

A strain gauge pressure sensor was employed by Xia et al. [15] to measure torque, with a strain amplifier and an Infinite Impulse Response low-pass filter being used to process the data. The results of the continuous measurement of soil mechanical resistance were found to be highly correlated with the average soil compactness measured by a hardness tester at the same location. It was observed that soil mechanical resistance is concentrated in a low-frequency random signal around 2 Hz. In the power spectral density method, the fractal dimension was found to be close to 2, indicating that soil mechanical resistance is irregular and spatially variable. It represents a moderate variation with spatial correlation within a certain range.

Three types of sensors—electrical conductivity sensors, mechanical sensors, and γ-ray sensors—were integrated into a single system by Naderi-Boldaji [16] to assess the feasibility of detecting soil mechanical resistance. The electrical conductivity sensor was embedded within a pointed cylindrical body, which was fixed near the tip of the subsoiler. Field tests showed that the correlation between soil mechanical resistance and electrical conductivity was not significant. However, a stronger correlation was found between soil mechanical resistance and the ratio of electrical conductivity to soil moisture content. This indicates that soil mechanical resistance can be calculated by combining the measured values of soil moisture content and electrical conductivity. Additionally, the γ-ray sensor was found to be more effective in detecting soil texture.

Zeng et al. [17] mounted a resistive flexible thin-film pressure sensor on the tip of a deep tillage shovel to measure the cutting force applied by the shovel to the soil. The sensor’s readings were utilized to calibrate the contact parameters in a numerical simulation model. The study demonstrates that incorporating a layered soil structure into the PFC 3D soil-tool model can effectively replicate the presence of a hardpan layer in actual field conditions. The simulation results revealed that both the traction force and the area of soil disturbance increase linearly with the working depth. In other words, the deeper the tillage depth, the more significant the required traction and the higher the power consumption.

Naderi-Boldaji et al. [18] equipped a 25 mm diameter, a 45° conical probe with force sensors, dielectric sensors, and acoustic sensors. The dielectric sensors operate using the fringing field effect between two adjacent ring electrodes. During the probe’s penetration into the soil, passive acoustic emissions were measured at high frequencies ranging from 10 to 350 kHz using acoustic emission piezoelectric sensors. These acoustic emission sensors were installed inside the cone, close to the tip, to detect passive acoustic emissions generated by the interaction between the cone and the soil, as well as the movement of soil particles around the probe. The research indicated that soil moisture content affects both the frequency distribution of acoustic waves and the resistance encountered during penetration. This makes it possible to estimate soil volumetric water content with significantly less error than when using dielectric sensors alone. Furthermore, by combining the data from all three types of sensors, it is feasible to more accurately predict soil compaction, with the error being substantially lower than that of using a penetrometer by itself.

Zhao et al. [19] positioned an S-type force sensor between the tractor’s hitch and the shovel handle, while an ultrasonic distance sensor was installed on the frame. The force sensor measured the soil cutting resistance during the deep tillage shovel’s penetration into the ground. This resistance was transmitted via the shovel handle to the force sensor. Meanwhile, the ultrasonic distance sensor was employed to measure the distance from the hinge point of the plow handle to the ground, allowing for the calculation of the actual soil mechanical resistance.

Meng et al. [20], with the aim of achieving temperature compensation and expanding the measurement range, attached strain gauges in a balanced bridge configuration to the component’s surface. As the component underwent deformation under load, the metal foil within the strain gauges would stretch or compress, resulting in a change in resistance. During the strain effect, there was a linear relationship between the strain and the rate of resistance change. Using a specific measurement circuit, the soil mechanical resistance could be indirectly measured, with the absolute error between the measured value and the actual value ranging from 0.2 to 2.7 N.

In comparison to discontinuous measurement methods, the continuous measurement of soil mechanical resistance can reduce the time required for measurement points and the workload of measurement personnel. By installing continuous measurement equipment in agricultural machinery, data collection and tillage operations can be completed in real time, significantly improving operational efficiency. This method can also reduce the measurement error of plow pan distribution in large fields. Through dynamic and continuous acquisition of soil mechanical resistance changes caused by the spatial heterogeneity of the plow pan, it can completely present the change process of soil mechanics during tillage, ensuring the integrity of soil mechanical resistance data.

The data obtained from continuous measurement can reveal the spatial position information of the plow pan that cannot be obtained by discontinuous measurement methods, obtain the spatial distribution of soil mechanical resistance, and effectively reduce measurement errors. In subsoiling operations, the real-time and continuously collected soil mechanical resistance data can be fed back to the control system to achieve precise adjustment of the tillage depth.

## 3. Status of Tillage Depth Detection

At present, the measurement of tillage depth is mainly conducted through indirect methods using various sensors. Common sensor types for this purpose include ultrasonic sensors, tilt sensors, attitude sensors, and infrared optical sensors. However, most existing tillage depth detection systems rely on just one type of sensor, which can be susceptible to environmental influences and often results in lower accuracy. To address this, the integration of multiple sensors into a single system, known as multi-sensor fusion, can significantly enhance the accuracy of tillage depth measurements.

### 3.1. The Detection Method Using Ultrasonic Sensors

The detection method of ultrasonic sensors is based on the uniform transmission of sound waves. Sound waves with a specific oscillation frequency are generated by a signal generator and propagate through the air at a constant speed. Ultrasonic echoes are produced when these sound waves encounter impurities or interfaces and are then received by the signal receiver [21]. Therefore, the distance is obtained by calculating the time interval using the formula involving the speed of sound.

Tests and evaluations of the detection accuracy of ultrasonic sensors in different fields were conducted by Mouazen et al. [22] by mounting the sensors on the bottom of a vehicle frame. Compared to manual measurements, it was found that better detection accuracy was achieved by ultrasonic sensors in soft sandy loam soils, while the accuracy was lower in soils with cover crops.

A closed-loop automatic control system for cultivation depth was developed by Adamchuk et al. [23], in which the distance between the cultivator frame and the soil surface was measured using ultrasonic sensors, thereby obtaining the cultivation depth.

Topcon Agriculture [24] in Ireland designed the NORAC tillage depth detection system. Ultrasonic sensors are installed on four disc harrow groups to independently adjust the tillage depth of each harrow group according to surface conditions. The operating tractor is equipped with an automatic navigation and global positioning system. The tillage depth of the harrow groups is displayed in real time through a virtual terminal and transmitted to a remote server via network communication. The remote server can also issue tillage depth adjustment commands based on the machine’s current operating conditions to achieve remote monitoring.

In China, the application of ultrasonic sensors for tillage depth detection has been further extended and developed. Liu et al. [25] designed a ground-conforming measurement device for a subsoiler, which consists of an ultrasonic distance sensor and a ground-following mechanism. A four-bar linkage was implemented to ensure the ultrasonic sensor avoids inaccuracies or signal loss caused by weak reflected wave signals. Additionally, posture detection sensors were employed to monitor the distance between the subsoiler’s frame and the ground, thereby determining the penetration depth of the subsoiler’s shovel. The results demonstrate that, under tillage depths of 20 cm and 30 cm, the detection error of the tillage depth did not exceed 10%.

Wang [26] employed inclinometer sensors and ultrasonic sensors to derive tillage depth through posture calculation and Kalman filtering. The system achieved a tillage depth error rate of 2.8–6.8%. Moreover, they connected the sensors to the controller using a Modbus bus, which enhanced the real-time performance and scalability of the information acquisition terminal.

Zhou [27] employed ultrasonic sensors mounted on a subsoiling shovel attached to a tractor and transmitted data via a CAN bus. Calculations indicated that the resistance during subsoiling operations is primarily affected by two parameters: tillage depth and operational speed. The relationship was found to be cubic with respect to depth and quadratic with respect to speed. When the tractor’s speed was 6.3 km/h, in the depth range exceeding 0.4 m, the maximum relative error was 5.79%, and the model achieved an accuracy of over 90%. At a speed of 5.4 km/h, the maximum relative error between measured and estimated values decreased to 4.93%, and the model’s accuracy exceeded 95%. While the model demonstrated high accuracy, it has certain limitations and is mainly suitable for estimating tillage depth in subsoiling operations.

Jiang et al. [28] designed a subsoiling tillage depth detection device by integrating ultrasonic sensors based on the time-of-flight method and infrared sensors using the triangulation distance measurement method. The data collected by these sensors were processed using a Kalman filter fusion algorithm to eliminate noise. Experimental results showed that under smooth field conditions, the infrared sensor achieved better detection performance compared to the ultrasonic sensor. Conversely, in straw-covered fields, the ultrasonic sensor outperformed the infrared sensor. By integrating both types of sensors, the system achieved more accurate results under varying subsoiling conditions. The fusion-filtered data successfully and accurately detected tillage depth.

The research process of ultrasonic sensors represents a continuous progression toward enhancing the detection accuracy of tillage depth. It evolved from the simple utilization of ultrasonic waves to measure the distance to the soil surface. To address the issue of signal loss, the mechanical structure was modified by adopting a four-bar linkage mechanism. In terms of acquisition algorithms, the Kalman filter was employed to remove noise and improve data accuracy. Additionally, the integration of infrared sensors with ultrasonic sensors through filter fusion has enabled the system to adapt to a broader range of scenarios, including both smooth and obstacle-covered fields. Meanwhile, with the development of the Internet of Things, users can now obtain real-time tillage depth data, marking a significant advancement in the field.

### 3.2. The Detection Method Using Tilt Sensors

Tilt sensors, unlike other types, determine the position of an object by measuring changes in its three-dimensional angles. For accurate measurement, the sensor needs to be attached to the surface of the object to detect the angle of inclination relative to the horizontal plane. When used for detecting tillage depth, tilt sensors typically measure the angle changes on a mounting device or a sectional mechanism, and the actual depth is then calculated using a specific formula. Compared to depth measurement methods that use potentiometers, tilt sensors offer a more compact design and are easier to integrate into essential agricultural equipment. Moreover, tilt sensors generally have a much longer mechanical lifespan than potentiometers, making them a more durable choice for long-term use.

The detection of tillage depth using tilt angle sensors is mainly applied to rear-mounted suspended subsoiling implements. At present, compared with the mainstream tillage mode of traction operation in America and European countries, the research on tillage depth detection by tilt angle sensors is more extensive in countries with more hilly and mountainous areas such as China and Japan.

Suomi et al. [29] designed a tillage depth detection system to utilize ultrasonic sensors mounted at the front of the chassis and inclinometers installed on the connecting arm between the gauge wheel and the chassis. This system measured height and angle, and the depth was calculated based on the correlation between these two physical parameters. Experimental results indicated that the detection error of this system was within 10 mm.

Zhao et al. [30] created a detector that comprises a frame, a tilt sensor, and a sliding board designed to move in sync with the undulations of the surface. This setup measures tillage depth by sensing the relative angle between the frame and the tractor and then applying geometric modeling that incorporates both depth and angle.

Li et al. [31] introduced a measurement device where the tilt sensor is mounted on the linkage between the frame and the gauge wheel. The device calculates the tillage depth through angle measurements and subsequent calculations. The findings indicated that the accuracy of this method was within 6%.

Xie et al. [32] equipped a tractor’s lifting arm with a tilt sensor to monitor changes in its angle relative to the horizontal plane. The actual depth of tillage was determined by analyzing the linear relationship between the inclination of the lifting arm, the geometric parameters of the linkage mechanism, and the voltage readings.

Ding [33] used an accelerometer to measure the vertical angle of the deep tillage frame’s front arm before and after the shovel penetrates the soil. They developed a calculation model to determine the actual tillage depth and provided a detailed model for this purpose. Furthermore, they employed an industrial camera to monitor the operation of the deep tillage shovel and transmitted both the depth data and the monitoring images through a 4G network.

The tilt sensor-based tillage depth measurement devices mentioned in the above literature generally calculate tillage depth by deriving feedback angles from ground profiling mechanisms, which is effective for flat fields. However, when field surfaces contain debris such as crop residues or soil clods, detection accuracy is affected. Additionally, profiling mechanisms are bulky, making installation and operation inconvenient.

Yang et al. [34] introduced a technique for measuring the tillage depth of a tractor designed for hilly and mountainous terrains, featuring automatic body leveling. This method relied on left and right inclination sensors to control the extension and retraction of the telescopic beams of the hydraulic cylinders on either side, enabling single-sided or double-sided leveling to accurately determine the subsoiling depth.

Zhu et al. [35] integrated high-precision gyroscopes, accelerometers, high-performance microprocessors, and advanced dynamic solving with the Kalman dynamic filtering algorithm into the JY-61 module. This integration eliminated the previously required lengthy mathematical solving process when using attitude sensors, enabling direct measurement of subsoiling depth.

Another approach calculates tillage depth by measuring the horizontal tilt angle of the tractor’s rear suspension lifting arm, avoiding the impact of uneven fields on detection accuracy. Nevertheless, due to the multiple connecting rods between the tractor and the suspended unit, operators may adjust the lengths of the lifting rods and upper pull rods during actual operations, leading to changes in geometric parameters that require sensor re-calibration. This renders tilt-sensor-based tillage depth measurement devices insufficiently convenient.

Yin et al. [5] analyzed the motion trajectory of the traction tractor and mounted subsoiler during operation, establishing a tillage depth detection model for the tractor and subsoiler system. He proposed a tillage depth detection method and system based on the attitude estimation of the subsoiling unit. The system involves installing attitude sensors on the tractor’s rear suspension rod and mounted subsoiler, which output angular measurements for real-time calculation of subsoiling depth. This attitude-estimation-based detection system demonstrates strong anti-interference capabilities, being unaffected by ground vegetation, crop residues, and soil clods.

Meanwhile, Fang [36] proposed a method for measuring subsoiling depth using a dual-angle sensor setup. By placing angle sensors on both the lower pull rod and the subsoiling shovel frame, the angles of these components were used to cross-reference each other. This approach effectively measured subsoiling depth on flat ground, as well as on uphill and downhill slopes. The researchers conducted a comprehensive analysis to compare the attitude calculation methods based on rear suspension devices, quaternion attitude algorithms, complementary filtering attitude algorithms, and Kalman filtering attitude algorithms. Through experimental verification and data analysis, the quaternion attitude calculation method was finally determined to be the most suitable solution for angle calculation. The system’s measured average depth was found to be within the acceptable error range as defined by the system design requirements, with the calculated average depth deviating by no more than 5% from the depth measured at five points across five zones.

### 3.3. The Detection Method Using an Encoder-Measuring Sensor

In the method of measuring tillage depth using an encoder, the encoder sensor is typically installed on the connecting shaft between the frame and the land wheel rocker arm, and the tillage depth is calculated by real-time acquisition of the rocker arm’s tilt angle data.

The encoder-based, adaptive, subsoiling depth detection system developed by Jia et al. [37] constructs an adaptive tillage depth monitoring system using an adjustable swing arm and encoder, which can select corresponding mathematical models according to different topographic conditions to achieve depth measurement in multi-terrain scenarios. A rocker arm structure with land wheels is set behind the subsoiling implement, and the encoder is used to measure the rotation angle of the rocker arm under different tillage depths. Field tests show that the system has strong topographic adaptability and can achieve precise measurement of tillage depth in multi-terrain conditions: The maximum measurement error on flat ground is 11.3 mm, and the error on sloping ground is −12.8 mm. The maximum relative errors of tillage depth measurement under different topographic conditions are 7.40% and 8.53%, verifying the adaptability of the tillage depth control system to the monitoring needs of complex environments.

### 3.4. The Detection Method Using Multi-Sensors

Kim et al. [38] created a real-time system for measuring tillage depth using a sensor fusion approach. The system includes a linear potentiometer, an inclination sensor, and an optical distance sensor. Additionally, a traction measurement system was developed with a six-component load cell, and its accuracy was confirmed at 98.9% through static load testing. The results from non-penetration soil tests showed that the sensor fusion of a linear potentiometer and an inclination sensor improved accuracy by 6.34–11.76% compared to the combination of an optical distance sensor and an inclination sensor. The analysis of experimental data including travel speed and traction indicated that tillage depth significantly affects accuracy, with a coefficient of determination of 0.847 at the low plow condition, a value relatively higher than that at the high plow condition.

### 3.5. Comparison of Different Tillage Depth Detection Methods

Although the depth detection of subsoiling has basically achieved automation, with significant improvements in accuracy, real-time performance, and stability, several limitations still exist in practical applications. We compared different tillage depth detection methods as shown in Table 2. When ultrasonic sensors are employed for tillage depth detection, they are susceptible to interference from crop residues and implement vibrations. While measuring tillage depth indirectly through inclination sensors that monitor the angle of lifting arms or draw bars in the three-point hitch system is not affected by surface residue coverage or implement vibrations, this method requires the establishment of complex mathematical models based on the geometric relationships between the three-point hitch and agricultural implements. Moreover, these geometric relationships need to be recalculated when different implements are attached. The combination of encoders and ground wheel rocker arms for depth measurement is not affected by surface crop residues and can be flexibly applied across different agricultural implements. However, when operating on soft ground surfaces, the sinkage of the ground wheel may impact measurement accuracy. In the application of infrared optical sensors, although triangulation measurement is not affected by measurement timing and does not result in overestimation due to computer instruction delays, the inherent zero drift of infrared sensors causes data fluctuation. Furthermore, this method requires a certain level of field surface uniformity. For attitude sensors, while the detection accuracy is relatively stable with minimal interference from environmental factors such as weeds and rocks, and the structure is simple with convenient installation and debugging, there exists a certain degree of error during significant variations in tillage depth due to the non-linear relationship between the angles of the three-point linkage joints.

## 4. Status of Tillage Depth Control of the Whole Machine in Subsoiling

Due to the complexity and variability of the field environment, the regulation of tillage depth often needs to be adjusted many times to achieve stable and consistent results. Undulating terrain in the field, differences in soil texture, and differences in crop growth needs all put forward diverse requirements for ploughing depth. In order to cope with these challenges, researchers have begun to pay attention to the online regulation technology of tillage depth, and the current tillage depth control research mainly includes two methods: the whole machine tillage depth control and the tillage depth control of each row.

According to the different attachment status of the tractor during operation, the tillage machine can be divided into two types: suspended operation and traction operation. And the corresponding tillage depth control method is also different.

### 4.1. Suspended Machine Tillage Depth Control of Whole Machine in Subsoiling

The tillage machinery for suspended operation is mainly rotary tiller and moldboard plow. Most of the cultivated machinery in developed agricultural countries in Europe and the United States are combined soil preparation machines, which are mainly traction operations, and there are few studies on the regulation of ploughing depth of suspended working tools. The depth control of domestic suspended work tools is mainly realized by the hydraulic suspension lifting of the tractor, and the height control, position control, force control, slip rate control, torque control, multi-parameter comprehensive control, etc., are distinguished by the control mode.

#### 4.1.1. Height Control Method

Height control refers to the operation of the whole tool, the hydraulic system is in the “floating” position, and the depth limit wheel of the farm tool changes with the height of the ground and floats up and down freely. Height adjustment is suitable for the use of cultivated land in which the soil is relatively soft and uniform, but the disadvantage is obvious because the vertical force of the rear suspension farm tool to the ground is borne by the ground wheel, affects the traction performance of tractor, increases the fuel consumption of tractor obviously, and in the case that the soil is harder and the soil moisture is large, the depth limit wheel cannot play a very good role.

#### 4.1.2. Torque Control Method

Torque control converts torque into displacement through a torque sensor on the output shaft of the gearbox and feeds back to the suspension control part through the rod mechanism. When the joystick is fixed, the system automatically controls the output shaft torque to keep it within a set range. The torque control technology has a wide range of applications, which is not limited by the hook-up method, and has a wider range of application than traditional force regulation. However, torque transducers are complex and need to be installed in the drivetrain, which requires high reliability.

#### 4.1.3. Position Control Method

Position control is the signal received by the position sensor, by controlling the commutation and opening and closing of the proportional valve and controlling the tractor suspension’s upper and lower position to adjust the ploughing depth. Compared with height control, position control will have a certain effect on the weight gain effect of the driving wheel, and the uniformity of tillage depth is better when the tillage soil quality is different.

Zhang et al. [39] developed a hydraulic suspension system for tillage depth adjustment based on CAN communication, and after collecting the displacement signal, the electro-hydraulic proportional valve is controlled by adjusting the pwm signal duty cycle, so as to realize the online adjustment of the hydraulic system and better control the ploughing depth.

Nie et al. [40] used a potentiometer to detect the tillage depth signal, detected the angle of rotation of the lifting arm according to the change of the potentiometer resistance, controlled the forward and reverse rotation of the stepper motor, and then controlled the distributor for oil distribution, so as to realize the control of the hydraulic suspension system and the automatic control of the ploughing depth.

Due to the depth of placing, the suspension device is affected by the undulating swing of the tractor greatly, as it varies, and in the cultivated land with large undulations, the phenomenon of tilting back and forth may appear. The unevenness of the soil quality in tillage can cause the change of traction resistance, so that the engine load is uneven and the work is unstable. Position control is not suitable for the situation in which the soil condition is uneven and complex and the cultivated land is undulating.

#### 4.1.4. Slip Rate Control Method

Slip rate control refers to the management of the drive wheel speed and land preparation tractor tillage’s driving speed by a rotary encoder, and the lifting of the adjusting hydraulic suspension device is limited to a certain threshold and interval, thereby achieving the effect of controlling the ploughing depth. The slip rate is the ratio of the difference between the theoretical speed and the actual speed of the vehicle to the theoretical speed. The purpose of slip rate control is to change the working resistance of the tractor unit by adjusting the tillage depth, which can make the cultivated land undulating and uneven, make the sliding rate stable near the set value to improve the traction efficiency of the unit, and reach a relatively stable tillage depth.

Bai et al. [41,42] put forward a fuzzy PID control algorithm with the slip rate as the target value. A photoelectric encoder installed on the drive wheel tire was used to measure the speed of the drive wheel. For two-wheel drive tractors, the driving speed of the tractor was obtained through the speed of the non-driving wheel. For four-wheel drive tractors, the vehicle speed was measured by a low-speed radar sensor installed on the vehicle body. The slip rate was calculated based on the vehicle speed and the drive wheel speed. A comparative analysis was conducted on the responsiveness, anti-interference ability, and adaptability of the fuzzy PID algorithm, traditional PID control, and fuzzy control. The results verified that the fuzzy PID control algorithm had the advantages of fast response, small overshoot, and minimal static error.

The slip rate is greatly affected by soil moisture and soil quality, when the soil moisture of untillage subsoiling is larger and the slip rate is too large, which can cause the tractor to slip on the spot and cannot subloosen the operation, further causing the traction efficiency of tillage machinery and the operation efficiency and the tillage stability to become seriously reduced.

#### 4.1.5. Force Control Method

Force control is the management of the resistance signal according to a tensile pressure sensor, which adjusts ploughing depth by tillage resistance; if tillage resistance is larger, it can ensure the passage of the machine tool by reducing tillage depth; on the contrary, if the tillage resistance is smaller, the tillage depth can be increased appropriately. Force control improves the adhesion performance of the tractor, reduces the resistance of farm tools, and makes the engine work stably.

Chen et al. [43] proposed a fuzzy control algorithm to tackle the challenges of significant inertia and response delay in hydraulic suspension systems under the force-control mode. A force sensor was installed between the suspension device and the loading cylinder, while a position sensor was employed to monitor the rotational angle of the lifting arm. The positional data from the sensor were input into the controller of the fuzzy control algorithm. Through force adjustment and interference elimination tests, the response time was measured to be 0.6 s. Notably, this research was confined to laboratory-scale soil groove experiments.

Li et al. [44] also used the PID paste control algorithm to improve the response performance of the suspension system, and carried out field experiments, and measured the response time of the suspension system by changing the tillage depth to make the tillage force jump from 3 kN to 7 kN. The response time of the suspension system was 5 s, and the overshoot of the system was 25%. In the process of tillage machine operation, when the working resistance is small, the sensitivity of the automatic adjustment effect of the force-controlled ploughing depth will be reduced, and the soil is different due to factors such as different textures, different water content. When the soil situation is complex, tillage uniformity will be affected to a certain extent.

Due to the dynamic change of soil-specific resistance, in order to reduce the deviation between the actual resistance value and the target resistance value that the suspension mechanism bears and maintain the stability of the engine load, the system needs to continuously adjust the farming tool tillage depth, and in this process, the tillage depth causes a large fluctuation in the tillage depth. In the position control mode, the position of the implement relative to the tractor fuselage remains constant, and although the uniformity of the tillage depth is ensured, the traction will still be produced due to the spatial heterogeneity of the soil texture, and the engine load fluctuates.

#### 4.1.6. Force–Position-Integrated Control Method

As a well-researched and basic multi-parameter control strategy, force–position-integrated control effectively integrates the advantages of resistance control and position control and overcomes the inherent defects of both. In this control mode, the electronic control unit collects the signals of the force sensor and the angle sensor in real time, compares them with the preset target value, and calculates the corresponding deviation value. The driver can adjust the weight of resistance deviation and position deviation in the control system by setting the force–position synthesis coefficient, so as to realize the precise regulation of the farming depth of agricultural tools. This control not only improves the adaptability of the system, but also enhances the stability of the operation.

Xi et al. [45,46] proposed a comprehensive control mode of force and potential based on the comprehensive degree coefficient and used the BP neural network to participate in the adjustment of the comprehensive degree system. This model adopts the principle of nonlinear mapping and has a strong generalization ability, and the simulation test shows that the BP neural network has a good application value in the electro-hydraulic suspension system. The average relative error was 1.29%.

Lu et al. [47] designed a fuzzy controller, which uses the comprehensive degree coefficient to convert the comprehensive adjustment of the force and position into the control of ploughing depth, and carried out a fuzzy operation according to the difference between the target value and the actual value. In the response of the output control signal to carry out force control, the response time from 0 to 20 cm was less than 1.7 s, the maximum error of 20 cm was 1.7 s within ±1 cm, and the maximum error of 20 cm was within ±1 cm.

Shang et al. [48] used two control strategies for control: the switching method and the weighted coefficient method of the comprehensive adjustment of force and position. By establishing the mathematical model of the system, a fuzzy controller for ploughing depth was designed, and Simulink was used for simulation analysis. The results show that both methods achieved good control effects without overshoot, a steady-state error less than 5%, and a response time less than 1 s. Under 4000 N interference, both methods can return to steady state within 1 s, and the change in tillage depth is less than 40 mm. Among them, the switch switching method has a large reduction in tillage depth but is not sensitive to the change of resistance during shallow tillage. The weighted coefficient rule shows better overall performance. Studies have shown that the on/off switching method is suitable for less demanding systems, while the weighted coefficient method can be applied to more complex situations.

Lv et al. [49] established a force–position-integrated control system that automatically adjusts the comprehensive proportion coefficient. The actual traction resistance of the rear suspension system was obtained by the force sensor and the maximum traction resistance Ptop, which the engine can bear when not damaged, and the maximum value of the traction resistance Ptmax, which keeps the traction efficiency of the tractor in the higher range, and the error tillage depth was obtained from the position sensor, adjusted, and judged the comprehensive proportion coefficient. The results show that the system can protect the unit in a complex and changeable working environment, ensure the tillage quality, achieve the highest traction efficiency, and reduce fuel consumption compared with the fixed composite coefficient.

Although the force and position control system based on the fuzzy control algorithm can effectively control various nonlinear controlled objects and systems where various parameters are difficult to determine, the fuzzy control algorithm cannot effectively eliminate the static difference in the system. Cai et al. [50] proposed a P-fuzzy control scheme to solve the problem that the fuzzy system cannot eliminate, that is the static difference. Taking the absolute value of the ploughing depth deviation as the only basis of the control method, the integrator of the fuzzy control unit is used for the integral processing of the ploughing depth deviation, and the output is superimposed with the output of the two-dimensional controller, thereby eliminating the deviation of the fuzzy control system. The field depth control experiment shows that the P-fuzzy control scheme improves the response rate of the control system, and the P-fuzzy control scheme can also reduce the range of the fuzzy domain and make the system control’s accuracy higher.

Wang et al. [51] studied the comprehensive control of force potential based on soil-specific resistance in view of the working environment with large changes in soil conditions and explored the automatic control algorithm of its weight coefficient. Matlab was used to analyze the specific resistance of different soils, and the relationship between the weight coefficient and the specific resistance of soil was established, and an automatic control algorithm for the weight coefficient based on the soil-specific resistance was obtained. The variable weight comprehensive force and position control using this algorithm, which is hereinafter referred to as the “variable weight method”, was compared with the control mode featuring a weight coefficient of 0.5 through field tests. The results show that the variable weight method can ensure the tillage depth and give full play to the advantages of position adjustment in the area with small soil-specific resistance. In areas with high-soil-specific resistance, the performance of the engine can be ensured and the advantages of force regulation can be brought into play. This method can realize the automatic judgment and setting of weight coefficients in areas with large changes in soil resistance, which lays a foundation for a more accurate control of the system.

Wu et al. [52] proposed a variable synthesis coefficient control algorithm based on single-neuron PID, which automatically selects the appropriate synthesis coefficient through the monitoring of soil-specific resistance, and the control mode can realize the automatic adjustment of its coefficient according to the change of soil-specific resistance and conducts Simulink simulation experiments, which show that this method focuses on the uniformity of tillage depth when the tillage resistance is small, and gives full play to the advantages of position control, and can play the advantage of the traction control mode to ensure the stability of the engine load when the tillage resistance is large. In this way, the control problem caused by the change of soil-specific resistance can be solved well.

In the study by Fu et al. [53], in view of the situation of the suspended-tractor cross-area subsoiling operation, due to the change of soil texture, the tillage resistance involved changes, so that the value of the comprehensive control coefficient of the force level control cannot be determined. Based on the relationship between soil-specific resistance and the comprehensive coefficient, a method of automatic adjustment of the comprehensive coefficient by resistance type was designed. This method selects the corresponding comprehensive coefficient based on soil resistance, under the condition that the soil-specific resistance is large, satisfies the stability of ploughing depth, and when the resistance of ploughing depth is small, guarantees that the engine is relatively stable and does not damage the engine. The resistance self-adjustment model and the comprehensive coefficient of 0.5 were simulated and compared by Simulink. It was found that the two controls were similar in terms of tillage resistance at moderate tillage resistance. When the tillage resistance is small, the load condition of the resistance-type self-adjusting system engine is better, and the drag is not too large, and the tractor engine is damaged.

In a study by Chen et al. [54], in order to solve the problem of traction resistance and travel speed fluctuation and overshoot caused by the lag of the response of the electro-hydraulic suspension system during the ploughing operation of the tractor, the Smith estimation compensation control method was added to the ploughing depth control strategy, which eliminated the hysteresis link of the characteristic equation, so that the controlled quantity of the lag time was fed back to the output end of the controller in advance, and the controller issued instructions in advance to weaken the overshoot or oscillation caused by the time delay in the closed-loop loop of the electro-hydraulic suspension system. The results of ploughing experiments show that the average coefficient of variation of tillage depth of the fuzzy PID control strategy was 9.29%, which is 14.57% lower than that of the fuzzy PID algorithm, which can improve the uniformity of tillage depth according to the changed soil-specific resistance, the degree of concave and convex of the plot, and the traction resistance.

In the process of force position control, the cultivated land with undulating planting conditions in the plain area is mainly aimed at the cultivated land with a small spatial heterogeneity of planting conditions. However, when the tractor is operating in hilly and mountainous terrain conditions, the fuselage is tilted due to the terrain inclination, and the fuselage needs to be kept level by adjusting the lifting of the left and right wheels. This adjustment process can make the tool suspension change with the lateral angle of the tractor locomotive body and then cause the tillage depth of the left and right sides of the suspension device to be uneven, reducing the tillage accuracy and affecting the operation effect. The implement suspension system that the ordinary wheeled tractor is equipped with is difficult to adapt to the hilly and mountainous terrain, in which the lateral slope changes greatly, and cannot realize the effective lateral slope profiling function. Therefore, it is necessary to develop the implement suspension system with slope-adaptive performance.

Shao et al. [55] designed a hydraulic system of double hydraulic cylinders to adjust the transverse position and attitude of the three-point rear suspension mechanism of the medium-horsepower, hilly and mountainous tractor for the problem of transverse attitude adjustment, and determined the inclination angle and adjustment range of the rear suspension farm tool by the relative displacement relationship of the piston rod of the two hydraulic cylinders of the rear suspension mechanism of the hydraulic cylinder variable length lift bar tractor, and adopted the fuzzy PID control method to simulate and analyze the designed transverse posture adjustment of the hydraulic system and tractor rear suspension lateral attitude adjustment test. The results show that, when the tilt angle is set from 0° step to 15°, the system transition process time was 1 s, the system overshoot was 0, and the system control was stable to meet the lateral angle adjustment requirements of mountain and hilly operations.

Chen [56] designed an electro-hydraulic suspension system with a slope-adaptive adjustment function on its basis; the accelerometer and the magnetometer are installed at the center of mass of the tractor fuselage, and the two signals are fused through Kalman filtering to obtain the accurate estimate of the roll angle of the tractor body, and the fuzzy control algorithm is used to track the ideal roll angle of the electronically controlled hydraulic suspension at the same time, so as to adjust the comprehensive proportion coefficient of force level control and complete the slope-adaptive electro-hydraulic suspension’s roll angle control. The results show that the slope-adaptive electronically controlled hydraulic suspension can effectively track the lateral inclination signal of the slope, and the actual transverse depth fluctuation can be controlled within 10 mm.

Zhai et al. [57] used a fuzzy control strategy to carry out semi-physical (HIL) simulation analysis tests on terrain imitation control, force control, position control, and force–position comprehensive control for the construction of a tractor force model, suspension mechanism dynamics model, and whole machine dynamics model for the tillage operation conditions of tractors in hilly and mountainous areas. From the test results, it can be seen that the electro-hydraulic suspension control system of fuzzy PID has better performance, a higher response speed, and higher precision, and can realize the functions of imitation terrain control, force control, position control, and force–position comprehensive control, and meet the needs of hilly and mountainous tractor ploughing operations.

Compared with traditional force adjustment and position adjustment, the force–position-integrated control mode significantly enhances the system’s adaptability to terrain and improves the stability of subsoiling operations. In this control process, the above literature achieves collaborative adjustment of force–position parameters through the “switching method” and “weighted coefficient method”. Meanwhile, control algorithms are optimized and improved, such as introducing Smith predictive compensation control to eliminate the hysteresis link of the electro-hydraulic suspension system and adopting P-fuzzy control strategies, which further enhance the system’s dynamic response performance and control accuracy.

#### 4.1.7. Multi-Parameter Comprehensive Control Method

Multi-parameter comprehensive control refers to the most basic control mode, such as force adjustment, height adjustment, position adjustment, torque adjustment, and slip rate adjustment, as control factors, according to a plurality of basic control principles and multiple combinations to achieve a better depth control effect. For example, the comprehensive control of force position is to comprehensively consider the hanging position and tillage resistance to regulate the ploughing depth.

Tan et al. [58,59] adopted the slip rate of the tractor driving wheel, the traction resistance, and the farming depth of the farm tool as the control reference, studied the tractor electro-hydraulic suspension system in the way of comprehensive control strategy, and finally used the fuzzy control method to carry out the force and position control step response test of the electro-hydraulic suspension system. The experimental results showed that when the comprehensive weight was 0.6, compared with the simple force control and the position control results, it better reflected the stability of traction and the uniformity of tillage depth. It was feasible to use fuzzy control to get better results.

Zhang et al. [60,61] took the optimal slip rate as the control target and adopted the sliding mode variable structure control algorithm and the fuzzy PID control algorithm. According to the current system motion state and real-time feedback, the sliding mode variable structure control algorithm approached the sliding mode surface at a certain rate. In the approach process, both sides of the sliding mode surface in a specific way switched, the system state was forced to slide along the sliding mode surface, and the resistance of the system to parameter perturbation and external disturbance was enhanced, thereby improving the robustness and reliability of the control system. According to the field experiments, the average absolute deviation, maximum deviation, and variance of the slip rate were reduced by 27%, 49%, and 60%, respectively, when the sliding mode variable structure control method was applied. In addition, the variation of ploughing depth adjustment was reduced by about 27%, the displacement adjustment of the hydraulic cylinder was reduced by about 36%, and the variation of horizontal traction adjustment was reduced by about 42%, which significantly improved the operational stability of the tractor.

Yang et al. [62,63] proposed a tillage depth control method based on sliding mode variable structure control, which achieved precise tillage depth control by comprehensively adjusting the three parameters of resistance, position, and slip rate. In this method, the working principle of the comprehensive control of the force–position–slip rate was clarified in close combination with the actual ploughing conditions of the tractor, and a sliding mode variable structure controller with excellent performance was designed. Through field experiments, the results show that the method was reasonable and feasible compared with fuzzy PID control, which can adapt to complex tillage environment, meet the actual ploughing demand, and take into account the tillage quality and engine load, which provides an important reference for more accurate tillage depth control research.

Xu et al. [64] proposed a joint control method and control strategy for the comprehensive slip rate of force and potential and added the slip rate monitoring and control to adjust the tillage depth within the allowable range of the optimal slip rate range. The simulation model was established by AMEsim, and the slip rate and force synthesis fuzzy PID controller were designed, and the multi-parameter control effect based on the slip rate logic threshold was experimentally studied. The test results show that the system had a better response effect when the logic threshold of the slip rate was exceeded and had a better follow-up effect on the change of the slip rate.

Due to the imprecision of the sensor, the immaturity of hydraulic pressure, and control technology, the ploughing depth control technology of the agricultural tractor is controlled by the cultivator through manual operation. The disadvantages of this regulation mode are very obvious; when each component and structure carry out work, friction heat will be generated and cause the hysteresis, deformation, and expansion and contraction of element and member, which thereby affects the accuracy and stability of ploughing depth regulation. At present, due to the continuous improvement of sensor accuracy, hydraulic technology, and control technology, the electro-hydraulic system replaces the manual control valve with an electric proportional hydraulic valve, and the manual control mechanism is replaced with a control panel, a controller, and various sensors. The electro-hydraulic system is easier and more comfortable for agricultural producers to operate in the adjustment process, adjusts the hydraulic system more quickly, and improves the accuracy and stability of subsoiling operations. At present, according to the different application scenarios and operation requirements in Table 3, the force–position comprehensive adjustment and multi-parameter adjustment are widely used and can be well adapted to the soil fluctuation and change of specific resistance and can obtain better ploughing depth under the condition of ensuring the working stability of the tractor.

In agricultural production, the whole machine tillage depth adjustment is of great significance for land preparation equipment. It can effectively adjust the tillage depth of the whole machine or a group of ploughing parts of the whole machine or the combination of multiple machines. Through this adjustment, it can meet the requirements for tillage depth under different soil conditions and crop planting needs, thus creating a good soil environment for crop growth.

However, in the actual field operation process, due to the difficult attainment of a flat ground surface, most of the agricultural land has different degrees of undulation, and in the process of undulation, the thickness of the bottom layer of the plough is also different, which leads to the soil resistance of the subsoil shovels being affected by the degree of factors such as the ground surface undulation. When multiple deep ploughing shovels are in operation, there is a problem that the tillage depth of the subsoil machine is unstable at the lateral level.

This lateral instability in tillage depth can directly lead to inconsistent soil conditions in the lateral distribution. It may lead to the possibility that the soil in some areas may be over-tilled, while the soil in other areas may be under-tilled, which not only affects the structure of the soil, but also may adversely affect the growth of crops in the subsequent planting process.

In order to improve the quality and efficiency of land preparation operations, the development of a subsoil machine with a dynamic per-row tillage depth regulation system is particularly necessary. The uniqueness of this subsoil machine is that it can independently regulate the tillage depth of each countersink shovel. By adopting the method of tillage depth regulation per row, the ploughing depth of each immersed shovel can be accurately controlled according to the soil conditions and operational requirements in actual agricultural production, thus avoiding undesired ploughing depths of some immersed shovels due to uniform regulation. This significantly improves the stability of the tillage depth between rows and makes the soil condition more uniform and consistent across the field, providing more favorable conditions for crop growth.

### 4.2. Traction-Type Machine Tillage Depth Control of Whole Machine in Subsoiling

The tractor only provides traction during the field operation of the tillage machine of traction-type operations, and the depth of ploughing is mainly controlled by the depth limit wheel on both sides of the machine. The depth limit wheel can be copied anywhere, which can effectively ensure the consistent ploughing depth, and the subsoiler generally adopts the traction operation mode. The combined land preparation machinery in agricultural developed countries such as Europe and the United States generally includes multiple tillage parts, such as subsoiling shovels, disc harrow sets, pressing rollers, etc. Foreign agricultural machinery enterprises have also developed joint tillage equipment with the function of measuring and controlling depth.

Through experimental research by John Deere Company of the United States, it has been found that precise control of the ploughing depth of the machine has an important impact on maintaining soil moisture and preparing a good seed bed. Due to the variability of the degree of soil compaction, the depth of each tillage component needs to be adjusted online according to the soil conditions during the operation. To this end, John Deere has developed the TruSet monitoring system [65], which installs a tillage depth sensor between the depth limiting wheel and the frame, between the frame and each group of working parts, and completes the independent adjustment of the tillage depth of each working part through the supporting hydraulic system according to the prescription diagram containing soil compaction information. At the same time, the TruSet monitoring system combines GPS positioning technology to generate a map of the depth of cultivation during the tillage process and share it with the Agrian service platform, which can be combined with yield, nutrient, and other information to provide reference for the next step of farmland management.

The American CASE company [66] has developed an AFS (Advanced Farming Systems) ploughing depth intelligent monitoring system; before the operation, the prescription diagram containing the predetermined ploughing depth information of the plot is input into the system, the tractor is loaded with a GPS positioning system, and when the machine goes to the corresponding position, the hydraulic system automatically adjusts the tool to reach the specified ploughing depth. A stroke detection sensor is installed in the cylinder to provide real-time access to changes in cylinder length, which is key to precise depth control.

Fox et al. [67] developed the “Clemson Intelligent Plow” by converting an existing four-row subsoiler into a variable-depth tillage platform, guided by soil prescription maps generated from a cone penetrometer measurement system. This platform enables adjustment, with the maximum tillage depth reaching 45 cm of the thickness of the plow pan indicated in the prescription map. During prescription-based adjustments, the system reduces tractor fuel consumption by 45% and promotes deeper root growth in cotton crops, resulting in a 96% increase in taproot length compared to no-till fields.

The combined soil preparation machine of HE-VA [68] adjusts the tillage depth of the whole machine by regulating the height of the press roller through a hydraulic system. The subsoiling components adopt a hydraulic-excitation-sourced self-excited vibration mode, and the working pressure of the excitation source is regulated by the control valve group of the hydraulic system. The control valve group is located on the main circuit of the hydraulic system and is composed of control valve blocks for regulating the height of the press roller and the pressure of the subsoiling component excitation source, enabling independent adjustment of the press roller height and the excitation source pressure of the subsoiling components.

The hydraulic-excitation-sourced self-excited vibration subsoiler of JAMES Company similarly realizes the regulation of the working pressure of the subsoiling single hydraulic cylinder through a control valve installed on the main circuit of the hydraulic system [69]. The excitation source pressure of the subsoiling components is uniformly regulated by the excitation source’s pressure control valve block of the subsoiling components to maintain stable tillage depth under different soil conditions.

In the above study, the realization of online regulation of tillage depth depends on the plough depth prescription map formed by fixed-point testing of soil information and big data analysis. In Europe and the United States, the agricultural information service system is perfect, and there are professional service companies such as the Climate Corporation to assist users in establishing a soil information database and forming a prescription map for land preparation operations [70], and the regulation of ploughing depth is mainly based on the prescription map.

However, the China company is not able to provide more systematic prescription operation information in a short period of time, and the research on the cultivation depth control method obtained by soil compaction information in real time is more in line with the national conditions.

The whole machine tillage depth control is the current mainstream tillage depth control mode, which is a tillage regulation mode suitable for large-scale and flat farmland. By uniformly adjusting the ploughing depth of the entire agricultural machinery, the ploughing depth in the tillage area is consistent. This control method is easy to operate and suitable for large-scale operations, especially in areas with flat terrain and relatively uniform soil conditions. However, the accuracy of the whole machine tillage depth control is limited, and in the face of drastic changes in local terrain and small-scale fields, such as in southern China, when agricultural machinery need to be turned multiple times to complete the tillage operation, it may lead to partial turning and uneven tillage depth in areas with excessive fluctuations, which affects the tillage quality.

In contrast, the tillage depth control method of each row is more suitable for farmland with complex terrain, uneven soil conditions, and small plots, which is more suitable for the current land management model in China. It can be precisely adjusted to the specific topography and soil conditions of each row, and the tillage depth of each row of agricultural machinery can be adjusted independently, ensuring consistent tillage quality. This control method combines advanced sensor technology, automated control systems, and intelligent algorithms to monitor and adjust the ploughing depth of each row in real time, significantly improving the accuracy and efficiency of farming. However, the cost of equipment for the regulation of each row of tillage depth is relatively high, which will increase the purchase and holding cost of agricultural producers. The maintenance of systems, agricultural implements, and sensors is also relatively complex, which limits their widespread application to a certain extent.

## 5. Status of Tillage Depth Control of Each Row in Subsoiling

In agricultural production, the adjustment of the tillage depth of the whole machine is of great significance for the land preparation equipment. It can carry out unified control of the whole machine or independent control of the single-row subsoiler shovel of the tillage depth of the subsoiler. Through this adjustment, different soil conditions and crop planting can be met to meet the requirements of tillage depth, so as to create a good soil environment for the growth of crops.

In the actual field operation process, there will be inconsistent situations, including the thickness of the bottom layer of the plough, and the tillage resistances are inconsistent in different plots or in the same plot, which leads to the actual depth of the self-excited vibrating subsoiler shovel varying with the change of tillage resistance, and this variation may also occur between adjacent subsoiler shovels.

This variation in tillage depth may result in the over-tilling of soils in some areas and under-tilling in others, which not only affects the structure of the soil, but can also adversely affect the growth of crops during subsequent planting.

In order to avoid an undesirable subsoiling depth, each row of subsoiling shovel of the subsoiler is dynamically adjusted according to soil conditions, which is very beneficial to the improvement of subsoiling quality. The advantage of this adjustment mode is that it can independently adjust the tillage depth of each sinking shovel to reach the ideal ploughing depth, thereby avoiding the ploughing depth situation that some sinking shovels do not expect due to uniform adjustment. This significantly improves the stability of the tillage depth between rows, makes the soil condition more uniform throughout the field, and provides more favorable conditions for the growth of crops.

In order to improve the tillage quality of the self-excited vibrating subsoiler, Wang et al. [71] developed an electro-hydraulic control system and obtained the horizontal inclination angle on a single flexible plough by horizontally installing two groups of inclination sensors on the deep tillage frame and the inclination frame of the pressing roller and obtained the ploughing depth data in real time online. In addition, the tillage depth data obtained from the detection were used for the ploughing depth control of a single row, mainly by adopting the hydraulic cylinder as the excitation source and adjusting the working pressure of the hydraulic cylinder in time according to the change of the ploughing depth, which avoids a situation in which the ploughing depth is too large or too small. It was found that, under different driving speeds, the ploughing depth between the rows and in the row was stable, and the non-ideal tillage depth and traction force were reduced, and better tillage quality was obtained.

Subsequently, Wang et al. [72] applied this technology to the multi-row subsoiler and realized the independent control of each row of the multi-row subsoiler through the expansion of the control system. The improved electro-hydraulic control system investigated the tillage depth and traction and power consumption of inter-row and intra-row variations under field conditions and compared it with the classical spring system, and when the vehicle speed increased from 4.2 km/h to 6.2 km/h, the coefficient of variation of the electro-hydraulic system relative to the spring system decreased by 17.59 percent and 34.1 percent. The electro-hydraulic control system has a better loosening effect and reduces the bulk density of the soil layer.

Wu [73] has developed a deep cultivator based on ultrasonic sensors and hydraulic drives that automatically monitors and controls the depth of tillage. It is able to independently monitor and control the depth of each deep shovel.

Lou et al. [74] developed an electro-hydraulic control system to ensure that each subsoiler shovel gets the desired depth every second. In order to improve the quality of the tillage layer, the ultrasonic sensor is used to independently detect the depth of each row of tillage layer and adjust it through the hydraulic system. In the field test, the errors between the ploughing depth measured by the ultrasonic sensor and the actual tillage depth were 8.35%, 8.1% and 8.38%, and the average stability coefficients of the tillage depth were 97.5%, 97.9% and 94.9%. Compared with the whole machine control, the application of this row tillage depth control is more conducive to each row of the single subsoiling shovel to obtain the required tillage depth, and the stability of each subsoiling shovel’s tillage depth was improved. The standard deviations of tillage depth control for each row and the tillage depth control of the whole machine were 38.31 and 51.52, respectively.

Each row of tillage depth control can eliminate the whole machine control because of the undulating and turning of more conditions, caused by a single subsoiling shovel, cannot solve the problem of stable tillage depth, improve the accuracy of each row of single subsoiling shovel, and then improve the overall ploughing depth stability in the subsoiler operation. But each row adjustment needs to increase the detection sensor and the tillage depth adjustment device on each subsoiling monomer, and the cost is higher, and the application in agricultural production is limited.

## 6. Development Summary and Prospects

Deep tillage technology has the ability to break the plow pan layer, enhance water penetration, and improve soil permeability and aeration. It provides substantial benefits in terms of soil enhancement and boosting crop yields, and it has been extensively utilized in developed agricultural nations across Europe and North America. In recent years, China has increasingly focused on deep tillage technology as a solution to the problem of deteriorating arable land quality. At present, the measurement of tillage depth in China mainly depends on manual sampling and assessment, which involves selecting a few spots in the field, digging, and physically measuring the soil. This approach is not only labor-intensive and inefficient but also highly prone to human error, leading to significant inaccuracies. Furthermore, during the operation of deep tillage machinery, it is not feasible to acquire real-time data on the depth of tillage. Operators must rely on their experience to make necessary adjustments, which makes it difficult to achieve precise control over the depth of tillage.

This issue is especially pronounced for self-excited vibration deep tillage machines, where the depth of tillage is influenced by the working pressure of the vibration source and soil resistance. Without the capability for online monitoring and control of tillage depth, ensuring the desired depth becomes even more challenging.

The primary goal of deep tillage is to break the plow pan. In China, the position of the plow pan varies significantly in the vertical direction due to differences in the frequency and location of compaction from farming equipment. Hanging tillage implements typically use the tractor’s hydraulic system to control the depth of tillage, focusing on achieving a consistent depth or tillage resistance. However, this approach often overlooks soil compaction and the specific location of the plow pan. Towed tillage equipment, meanwhile, relies heavily on manual adjustments for depth control, also aiming for a uniform depth. Given the varied conditions of farmland across China and the inconsistency of plow pan locations, adjusting the depth of tillage based on the plow pan’s position can significantly cut down on energy use and reduce overall tillage costs.

In terms of future application scenarios, China is a country with a vast territory and various types of cultivated land. For example, the Northeast Plain area in China has flat terrain and a relatively unified planting mode, similar to the farming methods in North America and European countries. Therefore, traction-type tillage and subsoiling can be used, and it is recommended to develop large-scale tillage depth control technology based on prescription maps. In southwestern China, the terrain is similar to that of Japan, South Korea, and other regions, with many hilly mountain terraces. In the farmland with undulating small plots, the degree of soil compaction varies relatively little between small plots. Farmers need to use agricultural machinery to make more turns and lift the suspension mechanism. Therefore, it is recommended to develop multi-parameter tillage depth control, such as comprehensive control of force, position, and slip rate, so that tillage machinery can work better in hilly and mountainous areas and achieve stable operation. In the Huang-Huai-Hai region of China, long-term farming and the aggravation of salinization have led to serious compaction of the plow layer. To ensure the precise breaking of the plow layer, it is recommended to develop real-time-detection tillage depth control technology. The position of the plow layer is accurately predicted through indicators such as soil mechanical resistance, soil compaction, and moisture content, so as to construct a plow layer model and realize small-scale on-site detection and regulation during the operation of the machinery.

In the process of soil mechanical resistance detection, tillage depth detection and tillage depth control in subsoiling operations and multi-parameter detection and control will become the future trend. In this process, many sensors will be used, and the parameters obtained from the sensors will be processed. Many measured parameters have non-linear relationships with soil mechanical resistance and tillage depth. In this process, AI technology is used for numerical fitting, which allows for the separation of noisy electrical signal data from sensors and more accurate prediction. In the existing tillage depth detection, ultrasonic sensors and tilt sensors may be affected by tractor vibrations and uneven fields, and external signal interference may cause inaccurate data fluctuations. In the future, AI technology and semantic segmentation may be used to segment and predict targets, and then the tillage depth may be obtained through algorithm models. In addition, within the prescription map, unmanned automatic tillage and subsoiling can be carried out according to the algorithm model.

As sensor technology advances, sensors such as pressure, strain, and conductivity have been utilized for the dynamic measurement of soil mechanical resistance. Researchers from around the world have explored methods for measuring soil mechanical resistance, showing that strain gauge pressure sensors can effectively capture soil resistance at multiple depth levels. However, these studies are currently limited to static or slow-moving conditions and have not yet achieved the ability to dynamically measure soil resistance or predict the position of the plow pan.

In developed agricultural nations, the emphasis is largely on large-scale, tractor-powered combined tillage machinery. These systems incorporate information such as soil compaction and historical crop yields to enable precise regulation of tillage depth. However, this approach depends on prescription maps for depth control, making it heavily reliant on sophisticated agricultural information systems. As of now, China has not developed a fully mature agricultural information service system, and the method of depth control using prescription maps is not yet feasible in the country. As a result, it is essential to explore new methods of tillage depth control that aligns with the specific conditions of Chinese agriculture and the detection of plow pan locations.

## Figures and Tables

**Table 1:** The characteristics of soil mechanical resistance detection method.

Soil Mechanical Resistance Detection Method	Sensor Type	Detecting Principle	Advantages	Disadvantages
Non-continuous Measurement	Force Sensor	The soil reaction force when the cutting blade is pressed into the soil	Offering high measurement accuracy in a static state	Inefficient and the discontinuous sampling data
Telemetry Measurement	Scanning Oscillator Waveguide	Microwaves are emitted to the soil sample through an angular antenna and measured by a waveguide detector after reflection.	Improved measurement speed, non-contact soil measurement, and lower damage to soil surface	Substantial external impact and unvalidated production feasibility
Indirect Measurement	Air Permeability Sensor, etc.	It detects soil air pressure and establishes a relationship between flow sensor and soil pressure.	Prediction of soil resistance by other parameters	Higher detection costs
Continuous Measurement	Strain Gauge Pressure Sensor	The tillage blade deforms when cutting soil, and the soil mechanical resistance is measured based on the deformation.	Simple structure, low cost, and high sensor accuracy	Requires high installation standards and accuracy degradation under long-term operation
	Octagonal Ring Force Sensor	This consists of several strain gauges forming an octagonal ring, with their resistance values characterizing soil mechanical resistance under force.	Measurement of forces and torques in horizontal and vertical axes	Composed of multiple force patches, measurement accuracy is directly affected by patch accuracy.
	γ-Ray Sensor	Detection is based on the absorption, reflection, or ionization excitation of γ-rays by the soil.	Strong penetration for excellent measurement of various parameters	Expensive, nuclear radiation hazards, and health risks to humans
	Flexible Film Pressure Sensor	It uses a flexible film sensor to detect the cutting force of the shovel tip on the soil.	Feasible for field operation, high sensitivity, fast response, and flexible installation	Not yet verified for other soil types
	S-Type Force Sensor	The S-type sensor is proportional to the external load, and resistance varies with load.	Low cost, easy to operate, low energy consumption, power-off data retention	Not particularly precise representation a general trend

**Table 2:** The characteristics of tillage depth detection methods.

Detection Methods	Measurement Principle	Measurement Parameters	Method Evaluation
Ultrasonic Sensor	It calculates the time difference between the emission and reception of ultrasonic echoes.	Time of wave propagation, height above the ground, and temperature	Vibration and debris in field affects measurement accuracy
Tilt Sensor	It establishes a relationship between the plowing depth and the inclination angle of the suspension arm.	Initial angle and real-time angle collected by the inclination sensor and length of the mechanical arm	Work in complex environments, high linearity and good stability, and requiring calibration before operation
Encoder	The encoder acquires the inclination angle of the swing arm in real time.	Initial angle and working angle and angle between the ground and the implement	Not affected by surface debris, applicable to slope and flat conditions, and low measurement accuracy occurs in soft surface soil

**Table 3:** Summary of the characteristics of traditional rear-mounted-suspension tillage depth adjustment methods.

Control Method	Control Factor	Control Principle	Advantages	Disadvantages	Applicable Scenarios
Height Control	Ground Wheel Profiling	The height of the depth-limiting wheel relative to the cultivated land	Good ground profiling ability and suitable for large ground undulations	High-soil-specific resistance, low tractor performance, and high fuel consumption	Uniform-soil-specific resistance and high requirements for weight
Force Control	Automatic Adjustment with Tractor Resistance	Good-weight-increasing effect and better plowing depth uniformity in different soil textures	Good-weight-increasing effect on the driving wheels and better plowing depth uniformity in different soil textures	Uneven plowing depth when the soil texture is non-uniform	Deep plowing operations and high soil mechanical resistance
Position Control	Setting Plowing Depth Value	Setting plowing depth value	Reduces the fluctuation range of traction force and suppresses the fluctuation range of plowing depth	Significant vibration impact and the engine load uneven with the change of traction resistance	Small resistance and flat ground
Slip Ratio Control	Determined by the Soil Moisture Content of the Land	The slip ratio is measured by the wheel speed sensor and the speed sensor.	Good traction performance and improves fuel utilization efficiency	High slip ratio lead to large fluctuations	Uniform soil moisture and moisture content with low slip ratio
Torque Control	Torque Sensor	The torque determined by the engine crankshaft linear velocity and the rated torque	Not limited by the hitch method and wide application range	Complex sensor structure and high self-precision and error increases with speed	High budget and low tractor running speed
Force–Position Integrated Control	Switching Method Weight Coefficient Method	Determining the value of the comprehensive weight coefficient	Reduces the fluctuation range of traction force and suppresses the fluctuation range of plowing depth	In high soil moisture, the tractor traction efficiency and tillage stability decrease.	Frequent soil-specific resistance fluctuations and the surface undulates
Multi-parameter Integrated Control	Multiple Sensors	Comprehensive intelligent control	Stable power output, high traction efficiency, and adaptable to different and heterogeneous soil textures	Complex control model	Multiple operation conditions and good adaptability

## Data Availability

Not applicable.
